# Geranylated 4-Phenylcoumarins Exhibit Anticancer Effects against Human Prostate Cancer Cells through Caspase-Independent Mechanism

**DOI:** 10.1371/journal.pone.0151472

**Published:** 2016-03-14

**Authors:** Noor Shahirah Suparji, Gomathi Chan, Hani Sapili, Norhafiza M. Arshad, Lionel L. A. In, Khalijah Awang, Noor Hasima Nagoor

**Affiliations:** 1 Institute of Biological Science (Genetics & Molecular Biology), Faculty of Science, University of Malaya, Kuala Lumpur, Malaysia; 2 Centre for Natural Product Research and Drug Discovery (CENAR), Department of Chemistry, Faculty of Science, University of Malaya, Kuala Lumpur, Malaysia; 3 Department of Biotechnology, Faculty of Applied Sciences, UCSI University, Kuala Lumpur, Malaysia; 4 Centre for Research in Biotechnology for Agriculture (CEBAR), University of Malaya, Kuala Lumpur, Malaysia; Swedish Neuroscience Institute, UNITED STATES

## Abstract

Geranylated 4-phenylcoumarins, DMDP-1 & -2 isolated from *Mesua elegans* were investigated for anticancer potential against human prostate cancer cells. Treatment with DMDP-1 & -2 resulted in cell death in a time and dose dependent manner in an MTT assay on all cancer cell lines tested with the exception of lung adenocarcinoma cells. DMDP-1 showed highest cytotoxic efficacy in PC-3 cells while DMDP-2 was most potent in DU 145 cells. Flow cytometry indicated that both coumarins were successful to induce programmed cell death after 24 h treatment. Elucidation on the mode-of-action via protein arrays and western blotting demonstrated death induced without any significant expressions of caspases, Bcl-2 family proteins and cleaved PARP, thus suggesting the involvement of caspase-independent pathways. In identifying autophagy, analysis of GFP-LC3 showed increased punctate in PC-3 cells pre-treated with CQ and treated with DMDP-1. In these cells decreased expression of autophagosome protein, p62 and cathepsin B further confirmed autophagy. In contrary, the DU 145 cells pre-treated with CQ and treated with DMDP-2 has reduced GFP-LC3 punctate although the number of cells with obvious GFP-LC3 puncta was significantly increased in the inhibitor-treated cells. The increase level of p62 suggested leakage of cathepsin B into the cytosol to trigger potential downstream death mediators. This correlated with increased expression of cathepsin B and reduced expression after treatment with its inhibitor, CA074. Also auto-degradation of calpain-2 upon treatment with DMDP-1 &-2 and its inhibitor alone, calpeptin compared with the combination treatment, further confirmed involvement of calpain-2 in PC-3 and DU 145 cells. Treatment with DMDP-1 & -2 also showed up-regulation of total and phosphorylated p53 levels in a time dependent manner. Hence, DMDP-1 & -2 showed ability to activate multiple death pathways involving autophagy, lysosomal and endoplasmic reticulum death proteins which could potentially be manipulated to develop anti-cancer therapy in apoptosis resistant cells.

## Introduction

Prostate cancer is the most common cancer as well as the second leading cause of cancer-related deaths in men [1]. Despite the availability of multiple treatment options, there are currently no effective therapies available for treatment of apoptotic-resistant androgen-independent prostate cancer which often arises after hormonal deprivation or ablation therapy [2]. Natural phytocompounds are considered as an important source of cancer chemopreventive and chemotherapeutic agents. Prominent examples include coumarin-based compounds which are derived from fruits and stem barks of various plants, such as *Casimora edulis* [3], *Calophyllum inophyllum* [4], *Mesua ferrea* [5] and *Mesua kunstleri* [6]. Coumarins have been recognized to possess anti-inflammatory, antioxidant, antiallergic, hepatoprotective, antithrombotic, antimicrobial, anti-arrythmic, anti-osteoporosis, antiviral, and anticarcinogenic activities [7–11]. Yang and colleagues, demonstrated fifteen isoprenylated coumarins isolated from *Mammea americana* exhibited significant cytotoxic effects and high anti-oxidant activity in human colon cancer cell lines [12]. In a study with both coumarin and 7-hydroxycoumarin, inhibition of cell growth in lung carcinoma cell lines by inducing G_1_ phase cell cycle arrest and apoptosis was demonstrated [13]. In another report, geranylated coumarins were seen to exert anti-proliferative actions through apoptotic cell death in leukemia cells [14].

In this study, two major geranylated 4-phenylcoumarins; DMDP-1 & -2 isolated from the bark of *Mesua elegans* (Clusiaceae), locally known as “pokok penaga”, were subjected to various cytotoxic and apoptotic assays. To the authors’ knowledge, this is the first report on the induction of multiple “apoptosis-like” caspase-independent programmed cell death on prostate cancer cells by geranylated 4-phenylcoumarins.

## Materials and Methods

### Collection of *Mesua elegans*

The bark of *Mesua elegans* (King) Kosterm was collected from Sungai Badak Forest Reserve, Kedah, Malaysia. The sample was identified by Mr Teo Leong Eng and deposited in the Department of Chemistry, Faculty of Science, University of Malaya herbarium (Ref. No: KL5232).

### Extraction and purification of coumarin analogues

**Fig 1:**
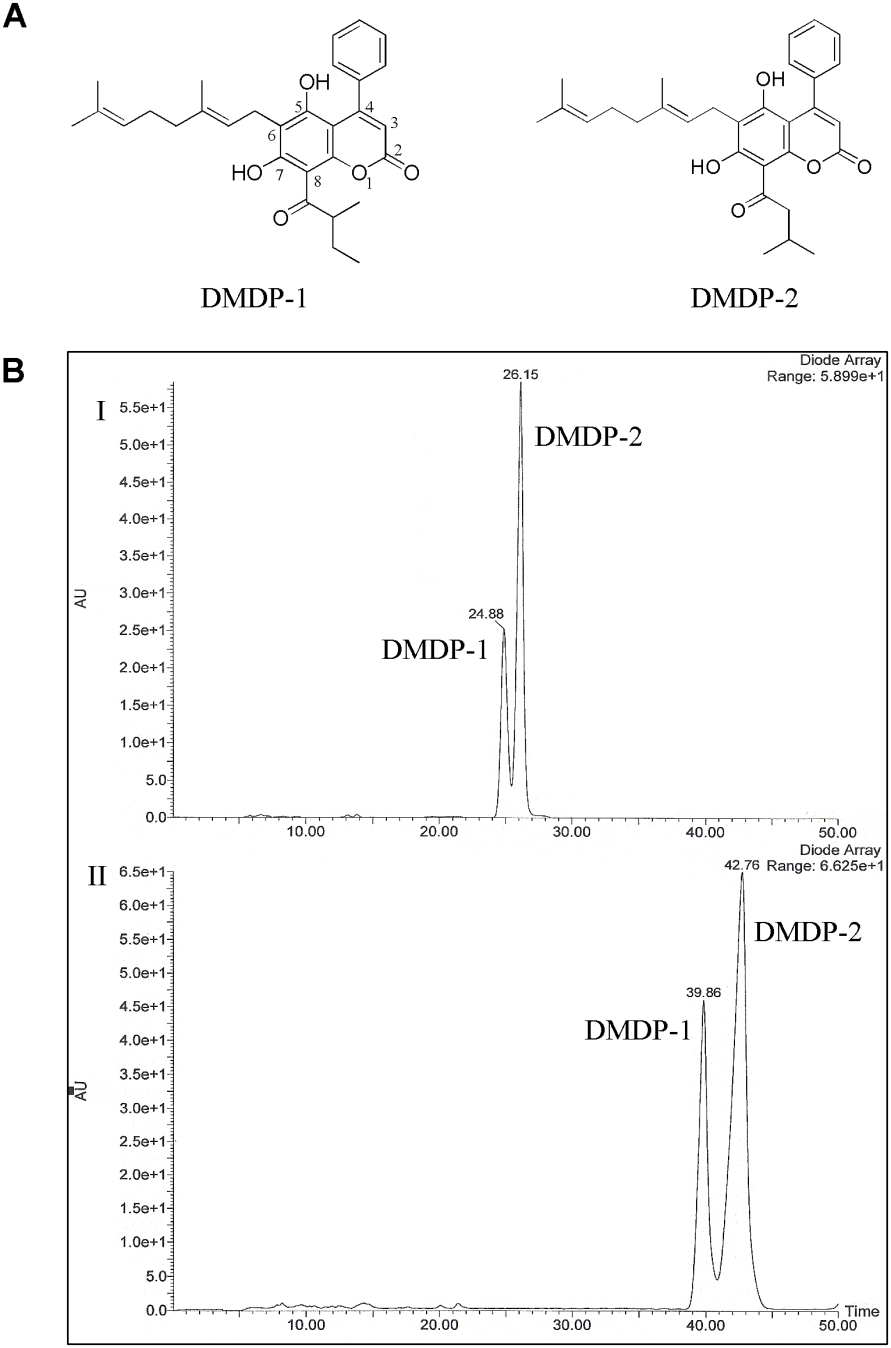
Isolation and characterization of two 4-phenylcoumarins from *Mesua elegans*. **(A)** Chemical structure of DMDP-1 & -2. **(B)** (I) HPLC chromatogram of fraction A2 with the following experimental conditions (analytical): column, ZORBAX Eclipse Plus C18, 4.6 mm i.d. x 150 mm x 3.5 μm; mobile phase, two solvents: A, 0.1% formic acid in H_2_0 and B, 0.1% formic acid in MeOH; the elution program at 0.6 mL/min as isocratic with 90% B (0–30 min) to afford isomers DMDP-1 & -2. (II) HPLC chromatogram of fraction A2 with the following experimental conditions (semi-preparative): column, ZORBAX Eclipse Plus C18, 9.4 mm i.d. x 250 mm x 3.5 μm; mobile phase, two solvents: A, 0.1% formic acid in H_2_0 and B, 0.1% formic acid in MeOH; the elution program at 3.0 mL/min as isocratic with 90% B (0–50 min) to afford isomers DMDP-1 & -2.

Dried ground bark of *Mesua elegans* (1.5 kg) was macerated with hexane (3 x 4L, 48 h each time) at room temperature. The extract was dried off using rotary-evaporator which yielded a yellow gummy residue (120.3 g). A portion of the crude hexane (13.0 g) was subjected to column chromatography fractionation over silica gel 60 (230–400 mesh) and eluted with hexane-EtOAc (from 9.5 to 0) and EtOAc-MeOH (from 5 to 0) to give fractions A-H. Fraction A was subjected to silica gel chromatography and eluted with hexane-EtOAc (from 9.7 to 9.5) to produce sub-fractions A1-A4. Observations of fraction separation were done using TLC with silica gel 60 F_254_ plates. Fraction A2 was subjected to HPLC analysis using ZORBAX Eclipse Plus C18, 4.6 mm i.d. x 150 mm x 3.5 μm HPLC column, and separated using ZORBAX Eclipse Plus C18, 9.4 mm i.d. x 250 mm x 3.5 μm HPLC column to purify isomers DMDP-1 & -2 (Fig 1). Water auto-purification system was used for HPLC separation. NMR spectra were obtained using JEOL LA400 FT-NMR and JEOL ECA400 FT-NMR Spectrometer System (400 MHz) with CDCl_3_ as solvent. UV spectra were recorded on a Shimadzu UV-Visible Recording Spectrophotometer using ethanol as solvent with mirror UV cell. The IR spectra were obtained through Perkin Elmer FT-IR Spectrometer Spectrum RX1 with CHCl_3_ as solvent. Mass spectra was carried out on Agilent Technologies 6530 Accurate-Mass Q-TOF LC-MS, with ZORBAX Eclipse XDB-C18 Rapid Resolution HT 4.6 mm i.d. x 50 mm x 1.8 μm column (S1 Table).

### Cell lines and culture conditions

A total of ten human cancer cell lines were used: Ca Ski, HeLa, HepG2, A549, PC-3, DU 145, MCF7 (purchased from ATCC), HSC4 (obtained from Cancer Research Initiative Foundation, Malaysia), MDA-MB-231 and SK-LU-1 (purchased from AseaCyte, Malaysia). NP69, the normal cell line was a gift from Prof GSW Tsao [15]. Each cell line was maintained with the appropriate growth medium (RPMI 1640: for cells A549, SK-LU-1, MDA-MB-231, PC-3, DU 145, MCF7) and (DMEM: for cells Ca Ski, HepG2, HeLa, HSC4) and (Keratinocyte-SFM: NP69), which were supplemented with 10% (v/v) fetal bovine serum. Cells were grown as monolayers at 37°C in humidified atmosphere with 5% CO_2_/ 95% air.

### MTT assay

The cytotoxic effects of DMDP-1 & -2 on cancer cell lines were determined using the MTT assay. A total of 1.0 x 10^4^ cells were plated (100μl/well) and treated at concentrations of 10 to 100 μM for 24 h with DMSO as a negative control. MTT solution (5 mg/ml) was added to each well and incubated at 37°C for 1 h. Absorbance was measured at 560 nm using a microplate reader (Tecan Sunrise^®^, Switzerland).

### Live/Dead assay

Assessment of cell viability upon treatment with both analogues was conducted using the LIVE/DEAD^®^ Viability/Cytotoxicity kit (Molecular Probes, Invitrogen, NY, USA). Cells were cultured on glass cover slip placed in 6-well plates and treated at IC_50_ concentration for 24 h. Cells were stained using a dual-fluorescence system consisting of 150.0 μl of calcein-AM (2.0 μM) and ethidium homodimer (EthD) (4.0 μM). Excitation and emission wavelengths were set at 494/517 nm for calcein-AM and 528/617 nm for EthD respectively. Visualization of samples was done using a Nikon Eclipse TS-100 fluorescence microscope (Nikon, Japan) under 100× magnification.

### Flow cytometer analysis

Detection of various cell death stages were conducted using Annexin V-FITC Apoptosis Detection Kit (Calbiochem, USA). Briefly, 1.0 x 10^4^ cells treated and untreated were incubated with Annexin-V (200μg/ml). Samples were centrifuged and re-suspended in 1× ice cold binding buffer with propidium iodide (PI). Detection and analysis was carried out using the BD FACSCanto II™ flow cytometer (Becton Dickenson, USA). For cell cycle analysis, 2.0 x 10^4^ cells treated and untreated were suspended in PI (50.0 μg/mL) and RNase A (10.0 mg/mL). These cells were fixed in ice-cold 70.0% (v/v) ethanol and kept at –20°C overnight. Cell cycle distribution was analyzed by flow cytometer and the percentages at different phases were determined by ModFit LT cell cycle analysis software.

### Apoptosis protein array

A total of 1.0 x 10^6^ cells were untreated and treated for 6 h. The cell lysates were normalized using BCA Protein Assay kit (Pierce, USA) and incubated at 4°C overnight with Raybio^®^ Human Apoptosis Antibody Array slides (RayBiotech Inc. USA, GA). All slides were washed and a cocktail of biotinylated antibody mix was used to detect apoptosis-related proteins. After incubation with Hylate Plus™-conjugated streptavidin, signals were detected with a fluorescence scanner by Axon Genepix^®^ using the Cy2 channel. Shortlisted protein expression target were selected based on a fold change threshold of ≥ 1.5 or ≤ -1.5.

### Western blotting

Nuclear and cytoplasmic proteins were extracted from PC-3 and DU 145 cell lines treated with analogues IC_50_ values for 6, 12, and 24 h using the NE-PER^®^ Nuclear and Cytoplasmic Extraction kit (Pierce, USA). Concentrations were determined using BCA Protein Assay kit (Pierce, USA). Equivalent amounts of protein (20 μg) from both untreated and treated cells were separated on 12% (w/v) SDS-polyacrylamide gels and transferred onto nitrocellulose membranes, which were subsequently incubated with primary antibody overnight at 4°C, followed by incubation with horseradish peroxidase (HRP)-linked secondary antibodies. 14 primary antibodies from Cell Signaling Technology, Danvers, MA against GADPH, caspase-3, -8, -9, PARP, Bcl-2, Bax, microtubule associated light chain (LC3), granzyme B, cathepsin B, calpain-2, p53, phospho p53 and p62/SQSTM1 were used. Protein bands were visualized via enhanced chemiluminescence signals on x-rays films. Intensities of all bands were quantified using image analysis software (NIH ImageJ v1.43, National Institutes of Health, USA). Anti-GADPH control antibodies were used for normalization of band intensities.

### Analysis of GFP-LC3

PC-3 and DU 145 cells were plated at a density of 4.0 x 10^4^ cells/well in 6-well plate, and left to adhere overnight in a 37°C incubator with 5% CO_2_. All cells were transduced with RFP-GFP-LC3B reagent using commercially available Premo Autophagy Tandem Sensor RFP-GFP-LC3B Kit (Life Technologies, USA) for 48 h. The following day, cells were incubated with 100 μM chloroquine diphosphate (CQ), and treated with either DMDP-1, DMDP-2 or left untreated (control) for 24 h. The cells were visualized under an inverted fluorescent microscope (Nikon Instruments, Japan) using a blue filter to detect the accumulation of GFP-LC3 punctate with GFP emission.

### Statistical analysis

All results were expressed as mean ±S.D of data obtained from at least three independent biological replicates. One-way ANOVA was employed to assess the significant differences between controls and treated samples with a *p*-value threshold of ≤ 0.05.

## Results

### Characterization of coumarin analogues

Both DMDP-1 & -2 were isolated from the hexane extract of the bark of *Mesua elegans* with >98% purity (S1 Fig). DMDP-1 was isolated as white crystals with m.p. 90–92°C while DMDP-2 as colorless oil. HRESIMS revealed an [M+H]^+^ ion peak at m/z 475.2728 (calculated 475.5945), corresponding to the molecular formula of C_30_H_34_O_5_ for both analogues. NMR, IR and UV data of these compounds have been studied and compared with literature [5, 16], and the structures of these compounds were confirmed as DMDP-1[5,7-dihydroxy-8-(2-methylbutanoyl)-6-[(*E*)-3,7-dimethylocta-2,6-dienyl]-4-phenyl-2*H*-chromen-2-one] and DMDP-2[5,7-dihydroxy-8-(3-methylbutanoyl)-6-[(*E*)-3,7-dimethylocta-2,6-dienyl]-4-phenyl-2*H*-chromen-2-one]

### Cytotoxic effects of coumarin analogues

**Fig 2:**
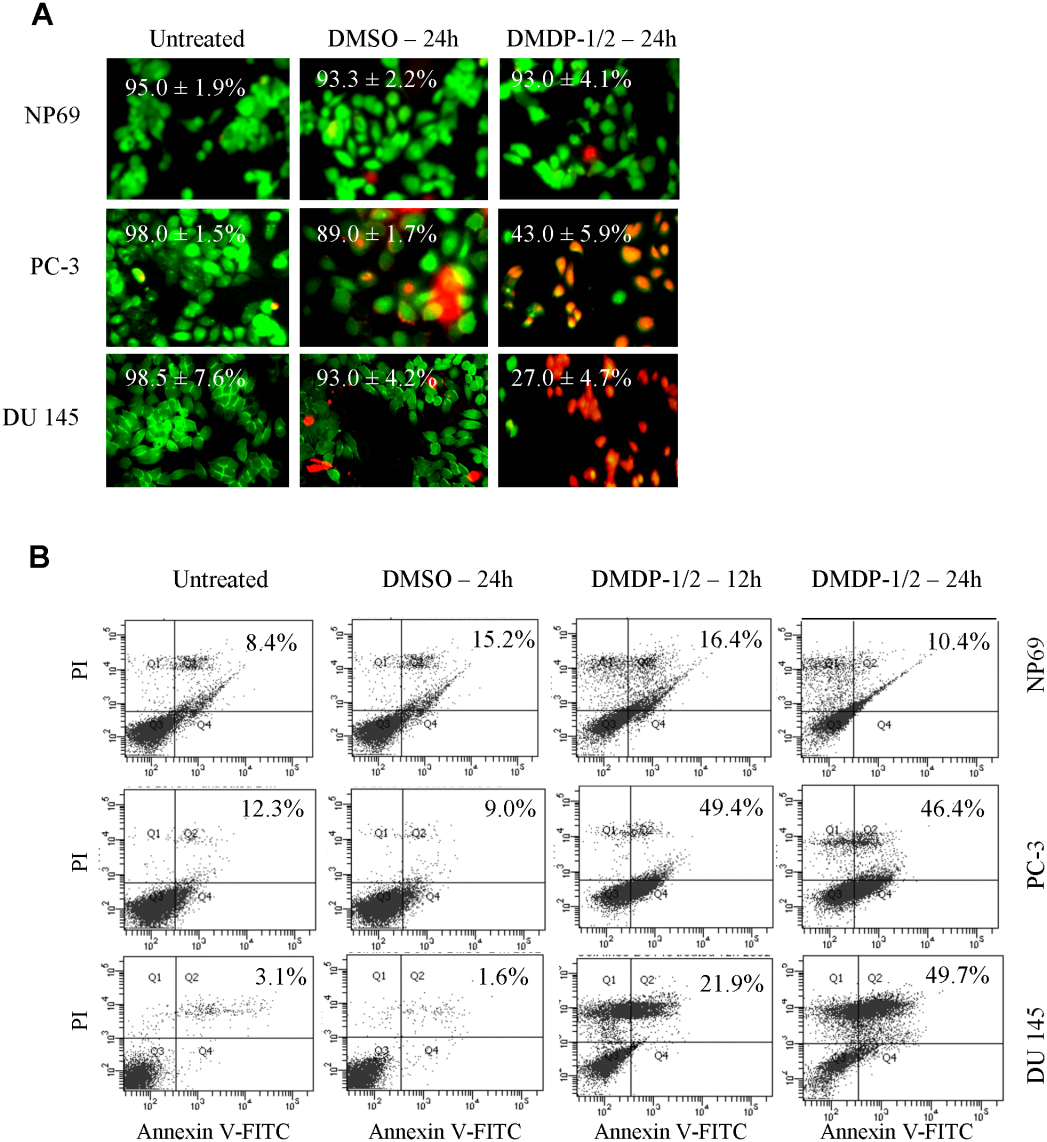
DMDP-1 & -2 induces cell death-mediated cytotoxicity in NP69, PC-3 and DU 145 cells. (A) Live/Dead assay after treatment with DMDP-1 & -2 for 24 h and DMSO as solvent control. Green fluorescence denotes viable cells stained with calcein-AM, while red fluorescence represents dead cells stained with ethidium homodimer. (B)Annexin V-FITC/PI flow cytometry dot plot analysis after treatment over 24 h. Percentage of cell death was calculated based on top right and bottom right quadrants from a total of 1.0 x 10^4^ cells.

Our results demonstrated induction of cytotoxicity in a dose and time dependent manner after 24 h of exposure on tested cell lines. Based on IC_50_ values, DMDP-1 induced cell death most effectively in PC-3 cells with an IC_50_ value of 9.0 μM while DMDP-2 performed best in DU 145 cells with a value of 24.0 μM, indicating a profound effect on prostate cancer cell lines (Table 1). Viability of cells treated with DMSO alone were insignificantly affected (≤2.0%) thereby ruling out the involvement of solvent-induced cytotoxicity. Live/Dead assays were performed to confirm their cytotoxic effects, cell death was only observed in PC-3 and DU 145 cells after treatment, but not in NP69 cells where viability was maintained at 92.7%. Percentage of PC-3 cell viability decreased from 98.0% to 43.0% while the viability of DU 145 cells decreased from 95.0% to 38.0% (Fig 2A). This indicated that both compounds inflict a more gradual cytotoxic pharmacokinetic effect in slow growing normal cells compared to rapidly proliferating cancer cells, which is a common characteristic among chemotherapeutic drugs.

**Table 1:** Summary of DMDP-1 & -2 mean IC_50_ values and mean viability levels obtained from MTT assays for ten human cancers and one normal cell lines after 24 h treatment. All values were presented as mean ±SD of three technical and three biological replicates.

		Mean IC_50_ ± SD (μM)		Mean Viability ± SD(%)^†^	
Human Cell Lines	Time (h)	DMDP-1	DMDP-2	DMDP-1	DMDP-2
Prostate adenocarcinoma (PC-3)	24	9 ± 1.2	38 ± 2.5	48 ± 5.1	34 ± 6.5
Prostate adenocarcinoma (DU 145)	24	45 ± 3.3	24 ± 1.6	22 ± 1.8	43 ± 2.9
Cervical carcinoma (Ca Ski)	24	47 ± 0.7	28 ± 3.6	40 ± 4.4	47 ± 8.4
Cervical carcinoma (HeLa)	24	n.d ^††^	81 ± 4.2	65 ± 5.5	46 ± 3.7
Oral squamous carcinoma (HSC4)	24	10 ± 2.5	32 ± 1.2	42 ± 3.5	44 ± 7.1
Breast adenocarcinoma (MCF7)	24	55 ± 2.1	60 ± 4.2	49 ± 3.1	51 ± 6.2
Breast adenocarcinoma (MDA-MB-231)	24	n.d ^††^	56 ± 1.1	86 ± 0.6	44 ± 0.4
Hepatocyte carcinoma (HepG2)	24	47 ± 2.2	59 ± 1.5	57 ± 4.6	46 ± 1.9
Lung adenocarcinoma (A549)	24	n.d ^††^	n.d ^††^	87 ± 2.7	80 ± 3.5
Lung adenocarcinoma (SK-LU-1)	24	n.d ^††^	n.d ^††^	64 ± 2.4	53 ± 0.7
Immortalized nasopharyngeal epithelial (NP69)	24	25 ± 3.0	38 ± 4.2	45 ± 9.4	25 ± 3.1

^†^Viability level of cells after treatment with 100 μM DMDP-1 or DMDP-2 for 24 h

^††^n.d. denotes an overall cell viability level of >50% after treatment with either DMDP-1 or DMDP-2 at 100.0μM for 24 h.

### DMDP-1 & -2 induces cell death

Following initial cytotoxicity data, we decided to focus this study on androgen-independent human prostate cancer cell lines. Annexin-V FITC/PI flow cytometry assays were used to determine mode of cell death. Cell death percentage was calculated based on top right (late apoptotic cells) and bottom right quadrants (early apoptotic cells) of the flow cytometry. The dot blot from a total of 1.0 x 10^4^ PC-3 cells after treatment with IC_50_ of DMDP-1 showed an increase from 12.3% to 46.4%. Similar effects were observed in DMDP-2 treated DU 145 cells where cell death population increased from 3.1% to 49.7% (Fig 2B). Even though cell death was also observed in NP69 cells, the cell death population was maintained below 10.4% after 24 h of treatment.

### DMDP-1 & -2 induces caspase-independent cell death

**Fig 3:**
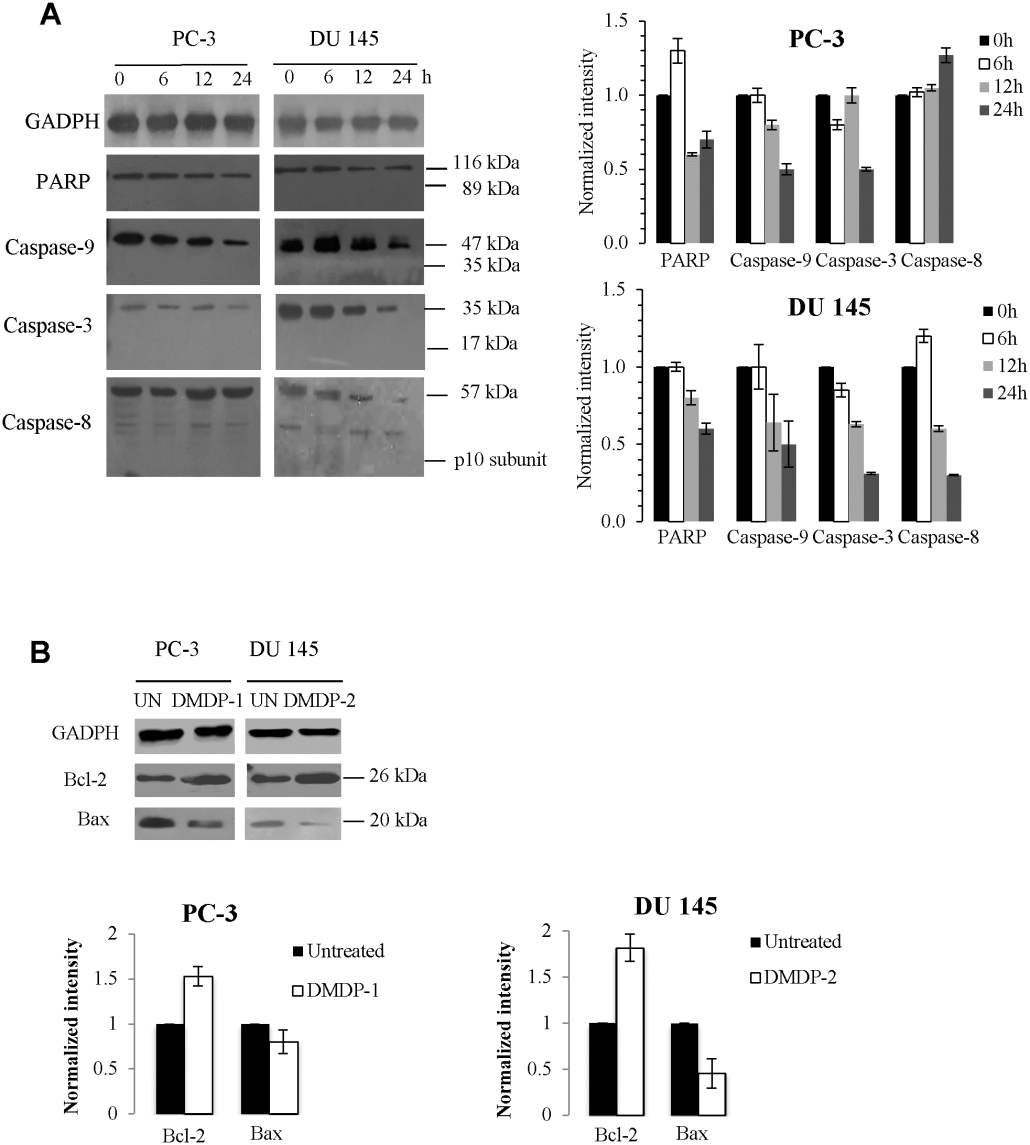
Western blotting confirmation on the effects of DMDP-1 & -2 treatment over 24 h on PC-3 and DU 145 caspase levels. (A) Treatment with DMDP-1 & -2 showed full length of protein levels and absence of cleaved PARP and caspases-9, -3 and -8. (B) Analysis of anti-apoptotic Bcl-2 and pro-apoptotic Bax protein levels. Quantification of protein band intensities were determined by densitometry analysis and normalized to GADPH using the ImageJ v1.43 software. All results were presented as mean normalized intensity ±S.D. of three independent experiments.

In our attempt to determine if the cell death was mediated via apoptosis, we used an antibody array capable of detecting 43 apoptosis-related genes involved in both intrinsic and extrinsic pathways. In Table 2, treatment of PC-3 cells with DMDP-1 demonstrated a reduced expression of survivin and insulin-growth factor receptor-1 (IGF1R) coupled with an increased level of insulin growth factor binding protein-2 (IGFBP-2) and heat-shock protein-27 (HSP-27). Meanwhile, treatment of DMDP-2 on DU 145 cells revealed reduced expressions of three proteins, that is, CD40-ligand, tumour necrosis factor receptor 1 (sTNF-R1) and TNF-related apoptosis-inducing ligand 1 (TRAIL-1). All other proteins tested did not show any significant change (fold change <1.5 or >-1.5) in expression, including prominent apoptotic effectors such as caspases-8 and -3, as well as Bcl-2 members such as Bad, Bax, Bim and Bcl-2 itself. Interestingly, this indicated that the death receptor pathway and the mitochondrial pathway were not activated following DMDP-1 & -2 treatments, but instead suggested a caspase-independent mode of cell death. In order to investigate the caspase-independent mechanisms involved, western blotting analysis of DMDP-1 & -2 treated PC-3 and DU 145 cells was carried out. The results was found to be consistent with data obtained from the protein array, where no induction of caspases-3, -8 and -9 proteins was observed, while the level of anti-apoptotic Bcl-2 was found to increase concomitantly with the change in Bax (Fig 3a & 3b). This observation was also consistent with the negative result obtained from poly-ADP ribose polymerase (PARP) cleavage, cell cycle arrest and DNA fragmentation assays where activation of caspases were found to be lacking (S2 Fig).

**Table 2:** List of significant protein level changes following treatment with DMDP-1 & -2 for 6 h on PC-3 and DU 145 cell lines respectively. A total of 42 apoptosis-related proteins were assayed using the RayBio^®^ Human Apoptosis Antibody Array G Series 1. Mean protein fold changes were calculated in comparison to untreated cells from four biological replicates.

**Protein Name**	**PC-3 treated with DMDP-1 (9μM)**	
	**Mean Fold Change ± S.D.**^†^	***p*-value** ^††^
IGFBP-2	1.7 ± 0.4	0.034
HSP-27	1.5 ± 0.5	0.036
IGF1R	-1.5 ± 0.2	0.012
Survivin	-1.6 ± 0.3	0.048
	**DU 145 treated with DMDP-2 (24μM)**	
	**Mean Fold Change ± S.D.**^†^	***p*-value** ^††^
CD40L	-1.5 ± 0.1	0.011
sTNF-R1	-1.5 ± 0.4	0.042
TRAILR-1	-1.5 ± 0.3	0.045

^†^Mean fold change threshold values were set at ≥1.5 or ≤-1.5

^††^*p*-value threshold value set at ≤0.05

### DMDP-1 & -2 induces cell death via multiple pathways

**Fig 4:**
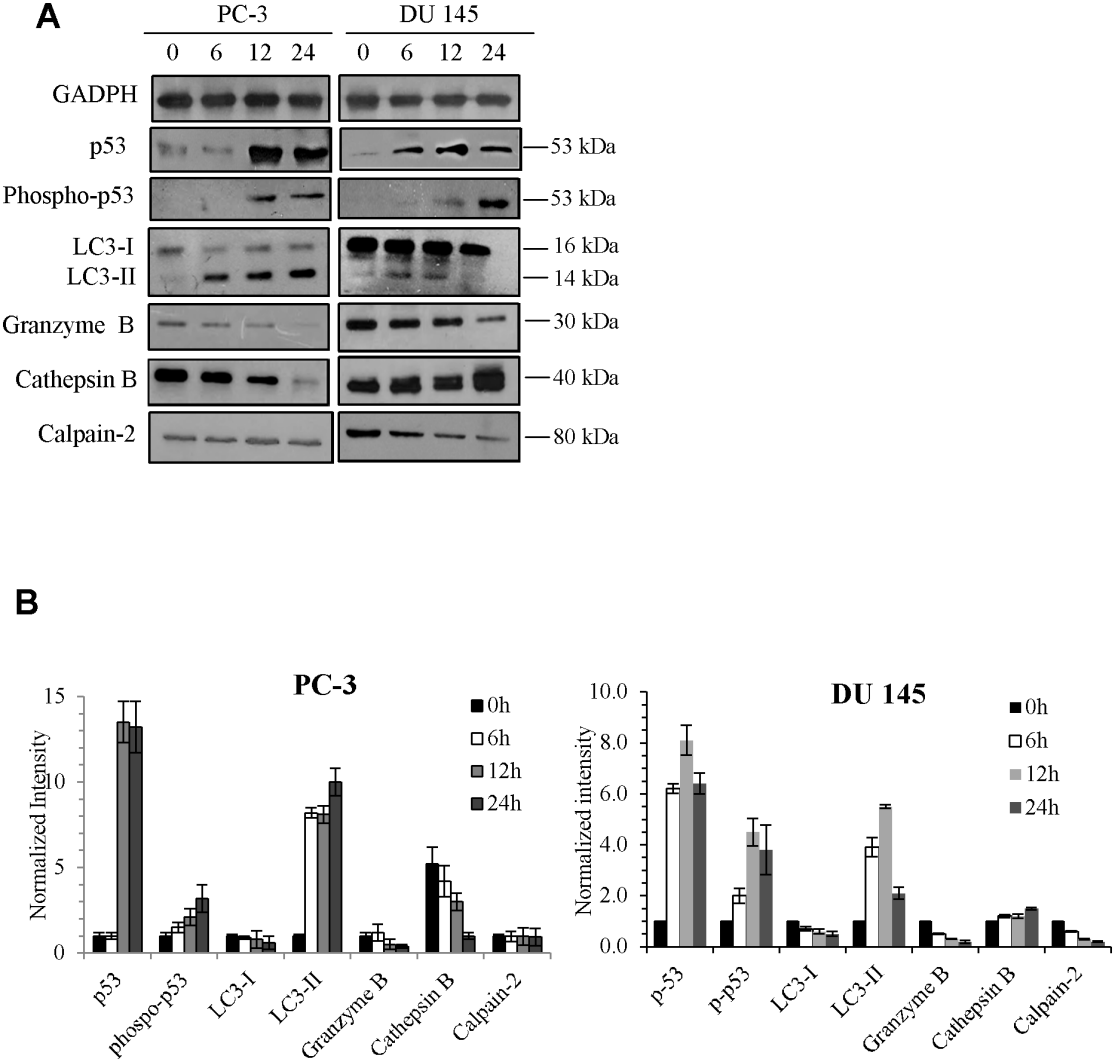
Activation of proteins involved in caspase-independent pathways. (A) Cells were treated with DMDP-1 & -2 over 24 h, followed by examination of key proteins involved in multiple mode of cell death by western blotting. (B) Quantification of band intensities were determined by densitometry analysis and normalized to GADPH using the ImageJ v1.43 software. All results were presented as mean normalized intensity ±S.D. of three independent experiments.

In order to identify which caspase-independent mechanisms were involved, western blotting analysis of treated and untreated PC-3 and DU 145 cells were carried out. In Fig 3, the absence of cleaved PARP, caspases-3, -8 and -9, up-regulation of Bcl-2 and down-regulation of Bax confirmed our hypothesis on the possible involvement of caspase-independent pathways mediated by autophagy or other organelles, such as lysosomes and endoplasmic reticulum (ER), which releases and activates death proteins such as, cathepsins, granzymes, and calpains. Western data (Fig 4) demonstrated an increase in cathepsin B levels in DU 145 cells which indicated that DMDP-2 induced the activation of this lysosomal cysteine protease in place of caspases. Calpain-2 levels, which are auto-degraded upon its activation[17], were decreased following treatment by DMDP-1 & -2. This result was further validated with combination treatment of each analogue with the calpain-2 inhibitor, calpeptin by western blot. However, granzyme B does not seem to play a role as its protein levels were reduced consistently over the 24 h course of treatment. Interestingly, the western results indicated that upon treatment, total and phosphorylated p53 levels were both up-regulated in a time dependent manner. Activation of p53 may increase the selectivity and safety of treatment with these two coumarin analogues by selective protection of normal cells and tissues.

### DMDP-1 induces autophagy and activates calpain-2 in PC-3 cells

**Fig 5:**
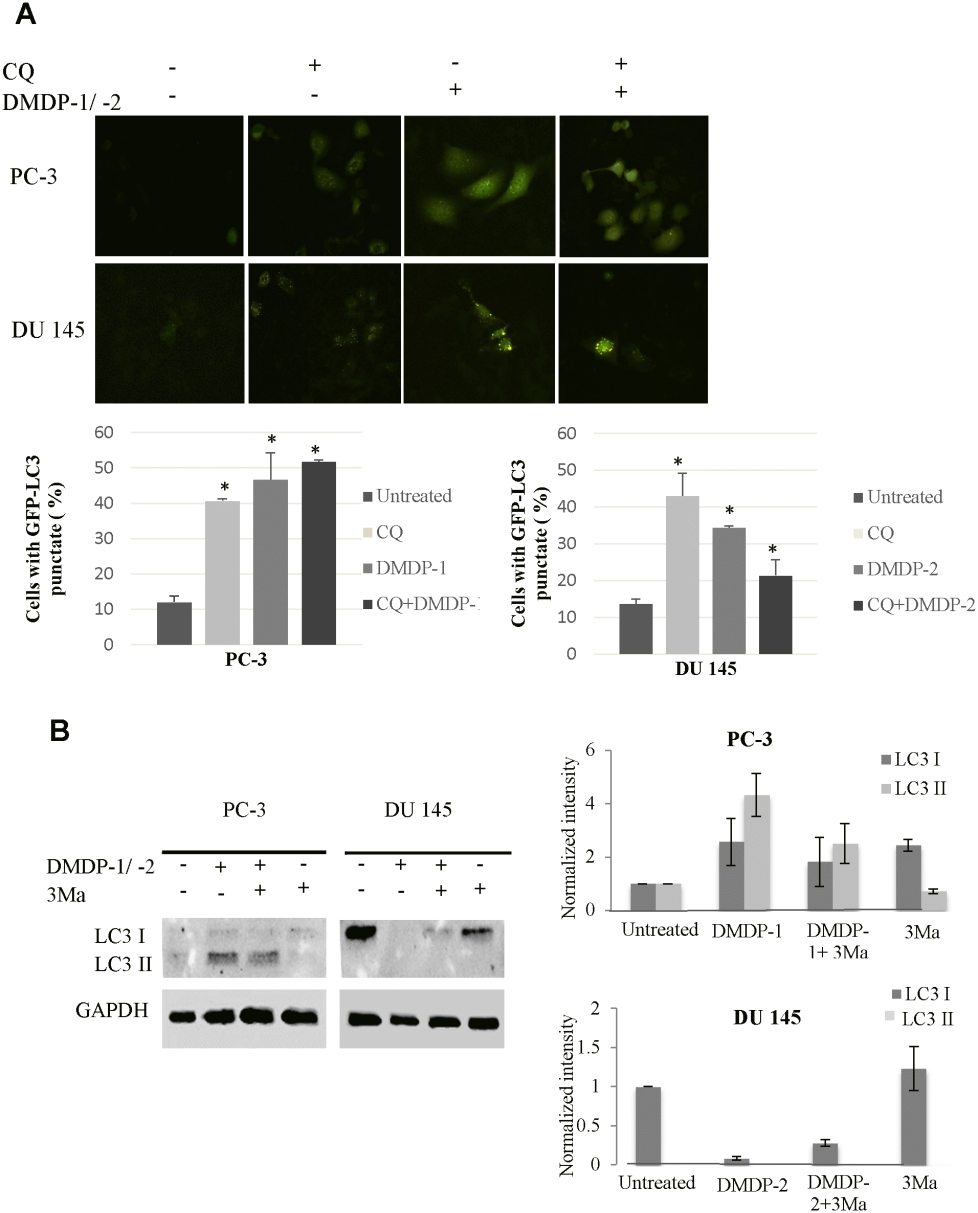
DMDP-1 induces autophagy. **(A)** Representative fluorescence images showing punctate distribution of GFP-LC3B in DMDP-1 & -2 treated cells. PC-3 in DU 145 cells were transduced with RFP-GFP-LC3B 48 h before treatment of 9 μM DMDP1, 100 μM choloquine or left untreated (control). Cells were also pretreated with 100 μM CQ for 4 h, followed by 9 μM of DMDP-1 & -2 treatment 24 h later, the punctate distribution of GFP-LC3 was visualized and compared with the diffused distribution in control cells. Percentage of PC-3 cells with obvious accumulation of GFP-LC3 punctate. Means ± SD, n = 3. *: p ≤ 0.05. (B) Expression of LC3-I/II treatment with both analogues with and without inhibitors for 24h. Quantification of band intensities were determined by densitometry analysis and normalized to GADPH using the ImageJ v1.43 software. All results were presented as mean normalized intensity ±S.D. of three independent experiments.

**Fig 6:**
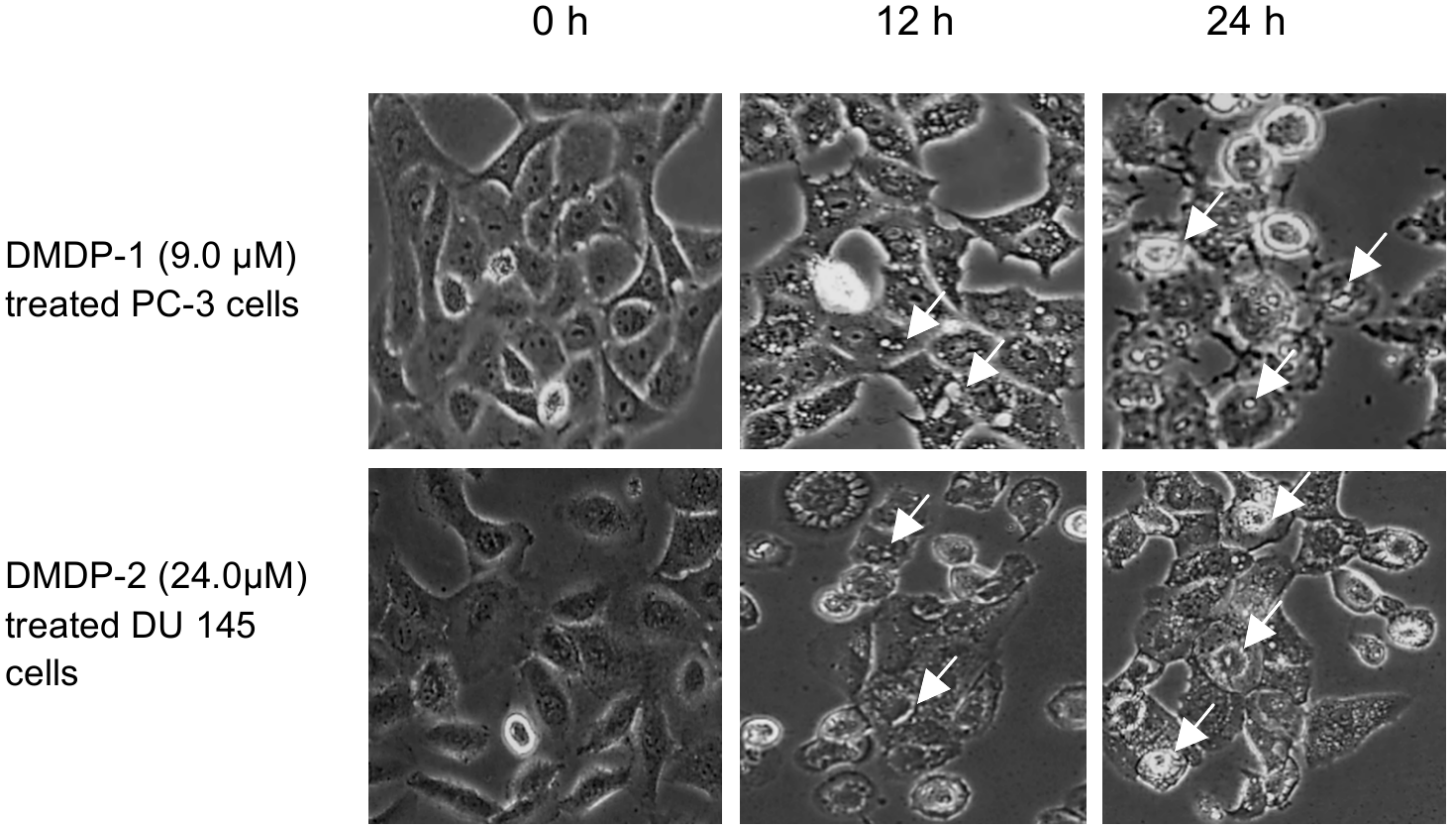
Photomicrograph of PC-3 and DU 145 cell lines depicting increased number of vacuoles after treatment with DMDP-1 & -2 for 24 h. All images are shown at 400x magnification, and formation of vacuoles is indicated by white arrows.

**Fig 7:**
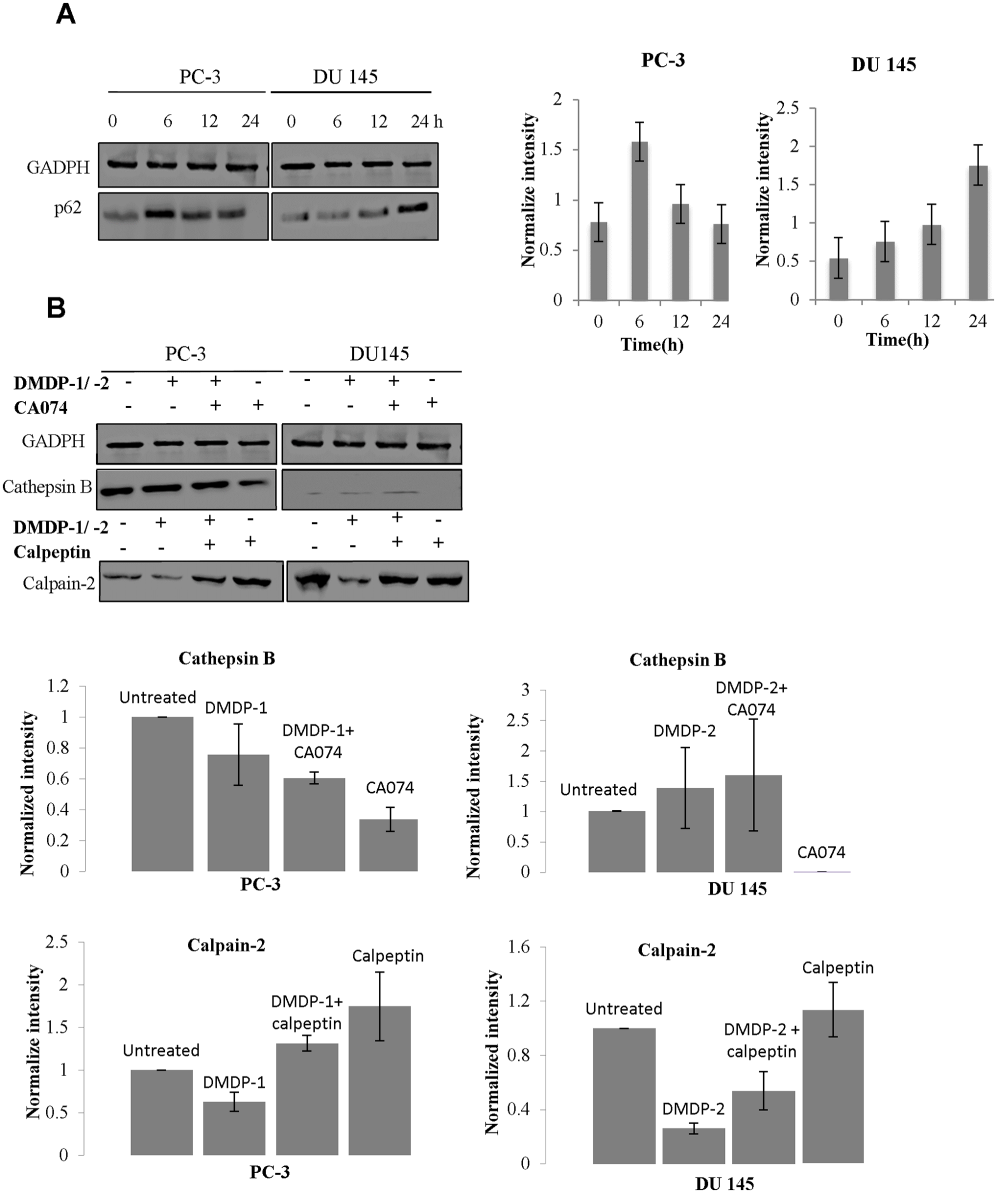
DMDP-2 activates lysosome, endoplasmic reticulum and inhibits autophagy. (A) Expression of p62 levels after treatment with both analogues on PC-3 and DU 145 at 0–24 h. (B) Expression of cathepsin B and calpain-2 treatment with both analogues with and without inhibitors for 24h. Quantification of band intensities were determined by densitometry analysis and normalized to GADPH using the ImageJ v1.43 software. All results were presented as mean normalized intensity ±S.D. of three independent experiments.

Western blotting on LC3-I/II protein level which is a standard marker for autophagy activation showed conversions of LC3-I to LC3-II after treatment with DMDP-1 and combined treatment of DMDP-1 and early autophagy inhibitor, 3MA in PC-3 cell line. However, no conversion was observed in the untreated and inhibitor-treated cells. Furthermore, analysis of GFP-LC3 result showed increased GFP-LC3 punctate in PC-3 cells pre-treated with chloroquine (CQ) and treated with DMDP-1, whereas the GFP-LC3 was distributed evenly throughout the cells in the control wells, suggesting a fusion event for autophagosomes and lysosomes in the DMDP-1 treated cells. GFP-LC3 punctate was prone to cluster and co-localize with lysosomes in cells treated with both CQ and DMDP-1. This is in contrary to that of DMDP-2-treated DU 145 cells, where minimal LC3-I/II conversion and reduced GFP-LC3 punctate even though the number of cells with obvious GFP-LC3 puncta was significantly increased in CQ treated cells (Fig 5). The formation of autophagosome was also seen as increased numbers of vacuoles in photomicrographs (Fig 6). In further investigating the effect of DMDP-1 & -2 on formation of autophagolysosome, the changes in levels of p62, a ubiquitin-binding protein (also known as SQSTM1) involved in autophagy, lysosome or proteasome-dependent degradation of proteins was observed. As shown in Fig 7A, treatment of DMDP-1 on PC-3 cells showed decreased p62 level at 24 h, providing further evidence that DMDP-1 induces autophagy. This result is in agreement with cathepsin B expression pattern, which decreased after treatment with DMDP-1 at 24 h (Fig 4). Meanwhile, in DU 145 cells, the increased level of p62 and cathepsin B suggested that DMDP-2 mediates lysosomal permeability resulting in leakage of cathepsin B into the cytosol to trigger downstream death mediators and not the fusion of autophagosome with lysosome to induce autophagy. Endoplasmic reticulum death protein calpain-2 activity in PC-3 cells was monitored with combination treatment of its inhibitor, calpeptin and DMDP-1 by western blot. Calpain-2 expression treated with DMDP-1 is the lowest in PC-3 cells as compared to the untreated cells, combination treatment of DMDP-1 with calpeptin and treatment with calpeptin only. Treatment with calpeptin only showed the highest calpain-2 expression as a result of inhibition of its auto-degradation, validating that calpain-2 activation in PC-3 cells is upon treatment with DMDP-1.

### Lysosomal and endoplasmic reticulum activity (ER) is involved in DMDP-2 induced cell death

Minimal conversion of LC3-I/II and increased expression of p62, strongly indicated that DMDP-2 inhibit a late stage autophagy, particularly at the point of lysosomal activity. As shown in Fig 7B, treatment of DMDP-2 in DU 145 cells for 24 hours resulted in increased expression of cathepsin B. Treatment with its inhibitor CA074 alone greatly reduced cathepsin B expression. A combination of treatment with its inhibitor, CA074 and DMDP-2 resulted in no significant change of cathepsin B expression as compared with the treated cells, confirming its involvement in cell death. Furthermore, we also measured the change in expression of the endoplasmic reticulum death protease calpain-2. Fig 7B shows a reduced protein level of calpain-2 as a result of protease activation and auto-degradation after treatment with DMDP-2 on DU 145 cells. A combination treatment of its inhibitor, calpeptin and DMDP-2 showed lower expression of calpain-2 as compared to the untreated cells but higher in cells treated with calpeptin alone, providing additional evidence of DMDP-2 induce ER cell death.

## Discussion

In terms of chemical structure, both geranylated 4-phenylcoumarins possesses the exact same characteristic coumarin ring, with only a slight variation; in DMDP-1, a 2-methylbutanoyl moiety is substituted at position C-8, whereby a 3-methylbultanoyl moiety at position C-8 in DMDP-2. The structure-activity relationship comparison showed that this single methyl substitution was capable of altering cytotoxic efficacies and preferences toward particular cell lines. This study provided evidence which supported a previous report that alterations in the chemical structure of coumarins could enhance their cytotoxic properties [11].

Activation of caspases have been frequently viewed as synonymous with apoptotic cell death [18]. However, cell death independent of caspase pathways are also important as safeguard mechanisms to protect the organism against unwanted and potential harm when caspase mediated routes fail, and can be triggered in response to cytotoxic agents or other death stimuli. Just as how mitochondria play a key role in apoptosis, other organelles such as lysosomes and endoplasmic reticulum also play an equally important role in the release and activation of death factors such as cathepsins, calpains, and other proteases [19–22]. Caspase-dependent and -independent apoptosis are all highly regulated forms of programmed cell death and play crucial roles in physiological processes such as tissue development, homeostasis and the elimination of unwanted cells [23]. Caspase-dependent pathway includes the extrinsic and intrinsic mitochondria pathways while caspase-independent, includes apoptosis-like, autophagic and necrosis-like cell-deaths mediated by lysosomes and ER [18, 19]. During apoptosis, caspase protein levels will not increase but will be processed by cleavage for activation while Bcl-2 proteins will be translocated to mitochondria. Report in 2000 by Saeki and his colleagues demonstrated that mechanisms other than apoptosis caused cell death in Bcl-2 down-regulated cells. Their finding indicated that the apoptosis inducing factor (AIF) did not translocate to nuclei as in the case of apoptosis, but rather it remained within mitochondria throughout the course of autophagy.[24].

Sometimes efforts to classify induction of specific programmed cell death, is difficult, as more than one death program may be activated simultaneously [25]. As evidenced in this study, a caspase-independent mode of cell death is induced from treatment with the coumarin analogues, and there are indications of involvement of not one but multiple apoptosis-like programmed cell death, namely autophagy, lysosome (cathepsin B) and endoplasmic reticulum (calpain-2).

Drugs that induces death through lysosomes and endoplasmic reticulum hold promise as targets and mediators of apoptotic signaling which may be less affected by intrinsic or chemotherapy-induced resistance mechanism [26]. Cancer cell lysosomes contain increased levels of cathepsins, and the release of these enzymes into the cytosol is considered to be the key activation step of the lysosomal death pathway [27]. Non-lysosomal calcium-dependent cysteine protease (calpain-2) is known as marker for ER cell death. Approximately 50μM of calcium ion (Ca^2+^) is required for its optimal activity in cells. Calpain-2 expression will be reduced as a result of auto-degradation upon its activation. Cancer cells often show evidence of constitutive ER stress, possibly due to hypoxia and glucose depletion which then leads to calcium influx in cells [26]. Various anti-cancer drugs, including cisplatin [28] and proteasome inhibitors [29], have been shown to induce ER stress.

Most cytotoxic agents trigger only the mitochondria pathway, however various agents such as paclitaxel [22], camptothecin [30], staurosporine [31, 32], also induced caspase-independent cell death mediated through cathepsin B/D and doxorubicin through calpains [33]. Therefore cellular death response triggered by cytotoxic compounds may not only involve caspases but proteases from other organelles which can act independently or in collaboration with each other. For example a “calpain-cathepsin cascade” has been reported, in which activated calpains induce release of cathepsins and subsequent cell death [34, 35].

This study also demonstrated that DMDP-1 induces autophagy while DMDP-2 inhibits autophagy flux via potential activation of the lysosomal death protein, cathepsin B and the endoplasmic reticulum death protein, calpain-2. Autophagy flux is defined as the complete process of autophagy beginning with formation of the autophagosome, followed by fusion of the autophagasome with the lysosome, and ending with degradation and recycling of the cell [36–38].

A well-accepted marker of autophagy is the vesicular accumulation of LC3 [39], due to the translocation of the modified LC3 from the cytoplasm to autophagosomes. The LC3 protein, which plays a critical role in autophagy, normally resides in the cytosol, but following cleavage and lipidation with phosphatidylethanolamine, LC3 associates with the phagophore and can be used as a general marker for autophagic membranes. We examined the effect of inhibiting the lysosomal turnover of autophagosome contents by CQ on DMDP-1 & -2 induced autophagy. CQ was used to artificially induce phagosome formation. As obvious punctate GFP-LC3 distribution is an indication for autophagosome formation, PC-3 and DU 145 cells were pretreated with 100 μM CQ for 4 h and then treated the cells with DMDP-1 & -2. Administration of CQ alone, blocked the fusion of autophagosome and lysosome; leading to accumulation of LC3-II formation and prevented degradation of p62 (SQSTM1). p62 is degraded at the end of the autophagy process by proteases in the lysosome upon the fusion of autophagosome with lysosome. Increase in p62 level showed that it was not degraded and resulted in an inhibition in late stage autophagy due to failure of autophagosome—lysosome fusion. Autophagy blockade is often associated with lysosomal membrane permeabilization and leads to cell death in the cells [38, 40]. Therefore, the decrease of p62 levels confirm DMDP-1 stimulated autophagy, while increased levels of p62 in DU 145 suggested DMDP-2 mediates lysosomal permeability resulting in leakage of cathepsin B into the cytosol to trigger downstream mediators of cell death and not the fusion of autophagosome with lysosome to induce autophagy. Thus, increased levels of p62 can be linked to the inhibition of autophagy [37, 38, 41].

DMDP-1 and -2 induces the formation of cytoplasmic vacuoles (Fig 6). Upon exposure to cytotoxic compounds, cells will attempt to sequester the drug into vacuoles to protect themselves. Vacuoles disappear after removal of the drug from the cell culture media, but prolonged vacuolization of lysosomes may lead to irreversible changes that result in cell death, particularly the release of cationic forms of the drug back into the cytosol. The most studied cytoplasmic vacuolation-induced cell death is autophagy. However, further study emphasized that cytoplasmic vacuolation-induced cell death has a physiological role and warrants further investigation. Many drugs, including chloroquine, neutral red, propranolol, atropine and lidocaine, induces cytoplasmic vacuolization [42, 43]

Understanding the potential of DMDP-1 and -2 in mediating multiple death modes, such as that of autophagy, lysosome- and endoplasmic reticulum- induced in this study, is crucial in further understanding the mechanism underlying carcinogenesis. This knowledge will provide the basis for newly targeted therapies, hence giving cancer researcher a better insight for future chemotherapeutic approaches.

## Conclusion

In conclusion, our present findings show that DMDP-1 induces autophagy and activates calpain-2, while DMDP-2 inhibits autophagy, activates cathepsin B and calpain-2, both leading to caspase-independent cell death in human prostate cancer cells. To the best of our knowledge, this is the first report on the induction of multiple caspase-independent death inducing pathways by naturally geranylated 4-phenylcoumarins, which has potential significant therapeutic implication on apoptotic resistant prostate cancer cells.

## Supporting Information

S1 Fig Chromatogram of purity percentage for DMDP-1 & -2 as obtained from HPLC analysis. Condition: column, ZORBAX Eclipse Plus C18, 4.6 mm i.d. x 150 mm x 3.5 μm; mobile phase, two solvents: A, 0.1% formic acid in H_2_0 and B, 0.1% formic acid in MeOH; the elution program at 0.6 mL/min as isocratic with 95% B (0–20 min).
(TIF)

S2 Fig Treatment of PC-3 and DU 145 cells with DMDP-1 & -2 respectively, indicating the absence of significant DNA laddering and cell cycle arrest. **(A)** DNA fragmentation assay after 24 h of treatment. Image acquisition was conducted using the Agilent 2200 Lab Tapestation. DNA marker (M: 50 to 2000 bps) and HL-60 cells treated with 0.5 mg/ml actinomycin D was used as positive controls (+). **(B)** Flow cytometer analysis for cell cycle arrest following 24 h treatment and subsequent propidium iodide staining on 2.0 x 10^4^ cells. DMSO was used as a solvent control in all experiments. **(C)** Positive control using paclitaxel for Annexin V-FITC/PI flow cytometry dot plot analysis after treatment over 24 h.
(TIF)

S1 Table ^1^H [δ_H_ (J in Hz)] and ^13^C (δ_C_) NMR spectroscopic data of DMDP-1 & -2 in CDCl_3_. (TIF)
