# Supplementary figures and images for: Geranylated 4-Phenylcoumarins Exhibit Anticancer Effects against Human Prostate Cancer Cells through Caspase-Independent Mechanism
Source: PLoS One. 2016 Mar 14;11(3):e0151472. doi: 10.1371/journal.pone.0151472 (PMC4790937; doi:10.1371/journal.pone.0151472)

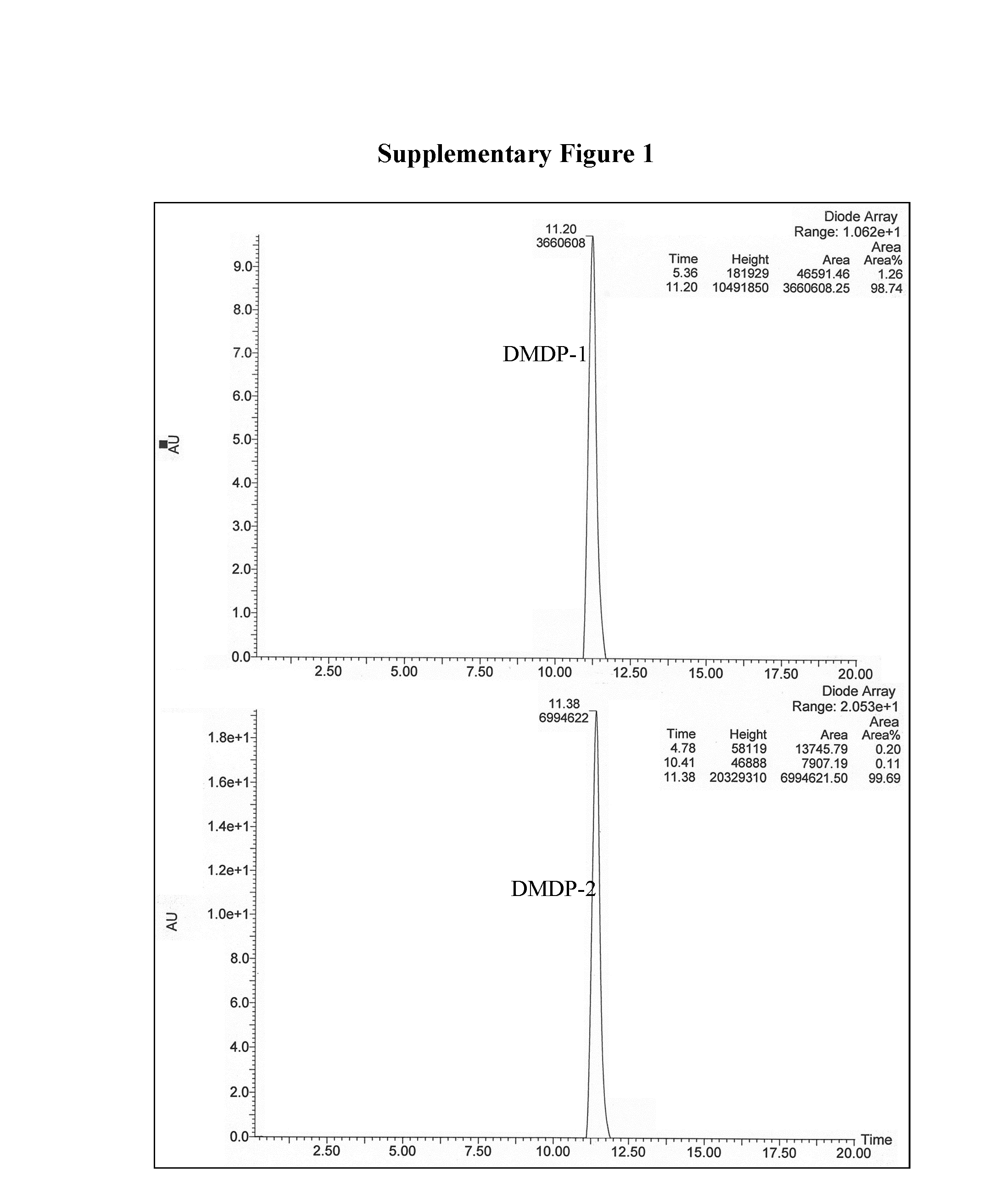

Supplement: S1 Fig — Condition: column, ZORBAX Eclipse Plus C18, 4.6 mm i.d. x 150 mm x 3.5 μm; mobile phase, two solvents: A, 0.1% formic acid in H20 and B, 0.1% formic acid in MeOH; the elution program at 0.6 mL/min as isocratic with 95% B (0–20 min). (TIF) [file pone.0151472.s001.tif]

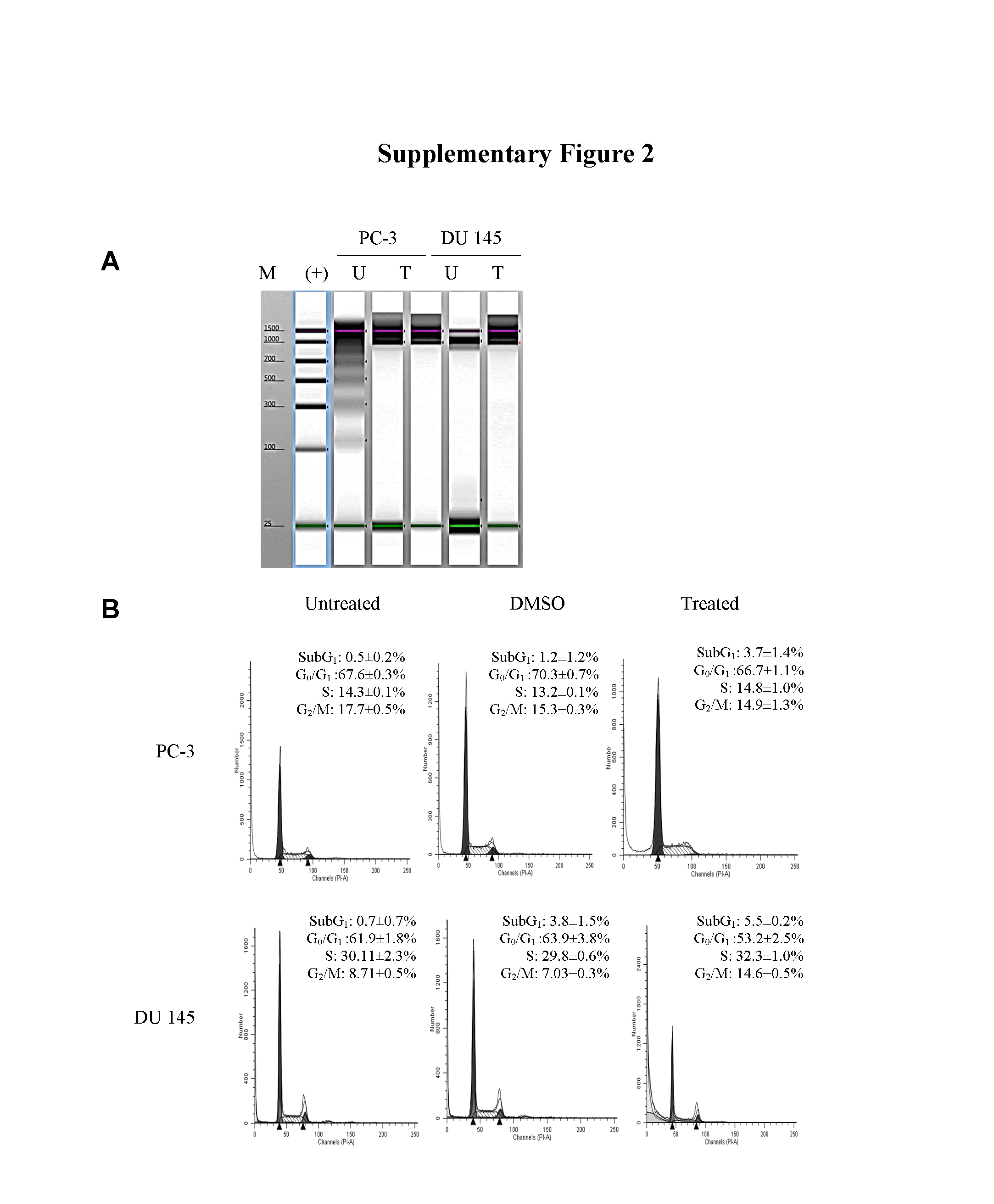

Supplement: S2 Fig — (A) DNA fragmentation assay after 24 h of treatment. Image acquisition was conducted using the Agilent 2200 Lab Tapestation. DNA marker (M: 50 to 2000 bps) and HL-60 cells treated with 0.5 mg/ml actinomycin D was used as positive controls (+). (B) Flow cytometer analysis for cell cycle arrest following 24 h treatment and subsequent propidium iodide staining on 2.0 x 10^4^ cells. DMSO was used as a solvent control in all experiments. (C) Positive control using paclitaxel for Annexin V-FITC/PI flow cytometry dot plot analysis after treatment over 24 h. (TIF) [file pone.0151472.s002.tif]

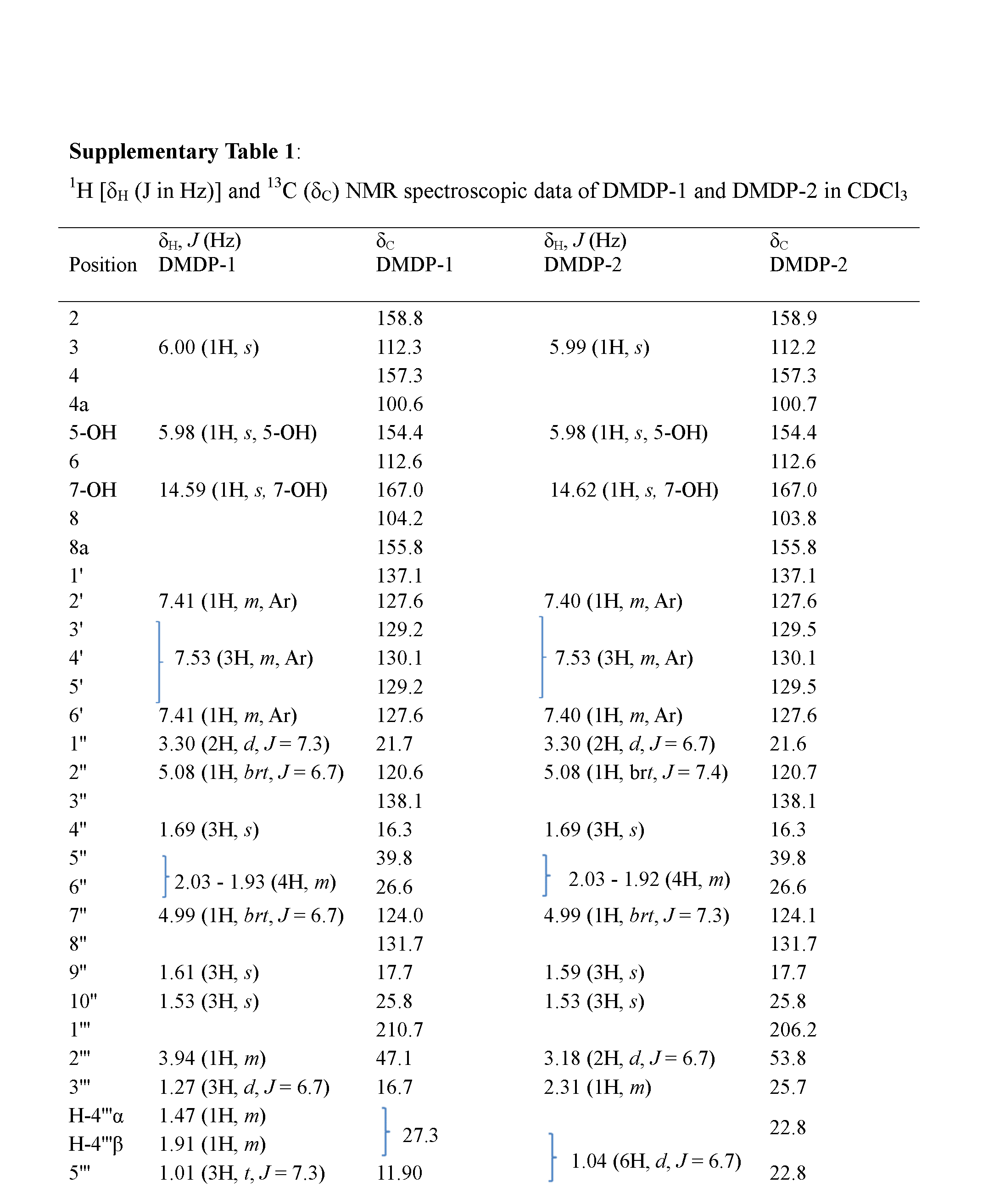

Supplement: S1 Table — (TIF) [file pone.0151472.s003.tif]
